# Prepectoral Breast Reconstruction without Acellular Dermal Matrix: Have We Come Full Circle?

**DOI:** 10.3390/jpm12101619

**Published:** 2022-10-01

**Authors:** Volker J. Schmidt, Navid Mohamadpour Toyserkani

**Affiliations:** Department of Plastic Surgery and Breast Surgery, Zealand University Hospital Roskilde, University of Copenhagen, 1165 Copenhagen, Denmark

Breast reconstruction is an integral part of breast cancer treatment [1]. Changing demographics, new technology, and increasing prophylactic mastectomies resulting in the need for bilateral breast reconstruction has increased the number of women undergoing implant-based reconstruction [2,3]. 

In general, breast reconstruction can be performed with autologous tissue or through implant-based reconstruction. It is overall believed that autologous tissue-based reconstruction results in a better quality of life than implant-based alternatives [4]. Autologous breast reconstruction has also been shown to be the most long-term economical option, demonstrating the greatest net monetary benefits [5]. However, not all patients are candidates for autologous tissue reconstruction, and there are also patients who decline autologous reconstruction due to concerns regarding donor site or the length of the operation. Other factors discouraging autologous breast reconstruction is that physician reimbursement per hour for breast reconstruction is much higher than it is for implant-based reconstruction if costs are not fully covered by the distinct healthcare system [6]. Further, patients often have to travel longer distances to receive autologous reconstruction compared to implant-based reconstruction [7,8] and microsurgical expertise and setup is lacking compared to the volume of patients in many regions [9]. For these reasons, implant-based options are a good alternative.

Implant-based reconstruction can be provided according to the direct-to-implant principle, in which a definitive implant is placed following mastectomy. Alternatively, a tissue expander that can be switched to a final implant in a later procedure can also be placed immediately following mastectomy. The disadvantages of tissue expansion include discomfort associated with filling the expander, injury to the expander, and a prolonged time to the final result [10]. Disadvantages related to immediate definitive implant placement include the inability to make finer adjustments to size, implant positioning, and increased stress to potentially compromised mastectomy skin flaps [10]. 

Historically, immediate breast reconstruction was synonymous with a two-stage approach using tissue expander implantation in the submuscular plane [11]. The advantages included better control over final implant position, breast size, and shape, and there was less risk of wound-healing complications. 

The advent of acellular dermal matrices (ADM) and other synthetic surgical meshes led to an evolution toward a dual-plane-like approach in which a mesh could cover the lower pole and act as an internal bra to support the implant. This would allow for a direct-to-implant approach and would thereby obviate the use of expander [12,13]. The direct-to-implant approach reduces operative stress for patients, and it has been shown to be cost effective compared to the two-stage expander procedure [14]. A multicenter study revealed that the direct-to-implant approach did not result in an increased rate of complications or adverse patient-reported outcomes [15]. Furthermore, the standardized intraoperative use of ICG-fluorescence imaging decreases mastectomy flap necrosis following implant reconstruction, and in particular, it makes direct-to-implant reconstruction more predictable regarding skin perfusion [16]. 

While the direct-to-implant approach carried out in a dual-plane fashion is traditionally described as using a supporting mesh for the lower pole, others have advocated for the same dual-plane approach without the mesh, achieving similar good results [17]. To avoid the use of mesh when possible is obviously more cost-effective and should be considered in a solidarity health system. In certain cases, due to compromised mastectomy flaps, an expander implant is used along with the dual-plane approach and mesh support; however, whether the mesh is really necessary when using an expander implant has recently been questioned, as a higher complication rate is seen without increased patient-reported outcomes [18].

Nowadays, there is a trend towards placing the implant+ADM in the prepectoral plane, which is also the most anatomically correct implant position to reconstruct the breast. Breast animation deformity is a major problem in submuscular implant placement and is something that almost all patients suffer from to some extent. It is also a major cause of decreased quality of life [19]. The prepectoral approach spares the underlying pectoralis major muscle and thus preserves the integrity and functionality of the chest wall musculature and circumvents the animation deformity problem. The prepectoral approach has generally not led to an increase in the complication rate [20]. The necessity of ADM for prepectoral expanders has likewise been questioned recently. Interestingly, no increased rate of complications has been reported when comparing prepectoral expander placement with and without ADM support [21,22,23]. 

One downside to prepectoral implant placement compared to at least an upper pole covered by the pectoral muscle is an increased incidence of rippling [24]. Rippling can be masked with fat grafting, and some even advocate for primary fat grafting at the time of implant placement [25,26].

There is a lack of robust data that highlight the benefits of surgical mesh devices in prepectoral breast reconstruction [10]. However, this is an important topic, as the acellular dermal matrix burden is associated with an increased risk of infection and device explantation secondary to infections [27]. 

With the latest developments, we have come full circle, as now, some studies advocate for the prepectoral placement of a definite implant without ADM, just as was originally described in the 1970s [28]. Some differences remain however, as there is now a greater emphasis on the preservation of mastectomy skin flap perfusion [29] and on flap perfusion evaluation methods as well as changes in implant characteristics [30]. 

As mentioned above, the introduction of indocyanine green for the evaluation of perfusion has drastically lowered the rate of complications related to suboptimal mastectomy flap perfusion [16]. This strong decision-making tool has helped fuel the evolution towards direct to implant principles and the prepectoral placement of implants. 

Furthermore, micropolyurethane-foam-coated implants have recently been proposed as an option for prepectoral direct-to-implant breast reconstruction [31,32] as they are proposed to provide mesh-like support without the need for further ADM manipulation, leading to pain reduction and decreased opioid usage [33]. 

When considering the choice of implant, the possible risk of Breast Implant-Associated Anaplastic Large Cell Lymphoma (BIA-ALCL) has to be taken into account. While the risk is relatively low, it has been suggested that the highest risk of BIA-ALCL is associated with the use of polyurethane implants [34]. Others believe that no conclusions can derived at this point in time and that further surveillance and research is needed before stating the increased risk of BIA-ALCL as a fact [35].

Technical advances have enabled better patient selection for prepectoral breast reconstruction. However, there is still a need for larger and better-quality studies that compare this technique with and without ADM before conclusions can be drawn. The evolution of implant-based breast reconstruction will continue and should always be centered on achieving the best possible outcome for patients at the lowest cost possible in order to be accessible for the different health care systems around the world.
